# Modelling the Ki67 Index in Synthetic HE-Stained Images Using Conditional StyleGAN Model

**DOI:** 10.3390/bioengineering12050476

**Published:** 2025-04-30

**Authors:** Lucia Piatriková, Katarína Tobiášová, Andrej Štefák, Dominika Petríková, Lukáš Plank, Ivan Cimrák

**Affiliations:** 1 Department of Software Technologies, Faculty of Management Science and Informatics, University of Žilina, 010 26 Žilina, Slovakia; dominika.petrikova@fri.uniza.sk (D.P.); ivan.cimrak@fri.uniza.sk (I.C.); 2 Department of Pathological Anatomy, Jessenius Faculty of Medicine in Martin, Comenius University Bratislava and University Hospital, 036 01 Martin, Slovakia; tobiasova11@uniba.sk (K.T.); stefak5@uniba.sk (A.Š.); lukas.plank@uniba.sk (L.P.)

**Keywords:** hematoxylin and eosin, Ki67, conditional GAN, StyleGAN, digital pathology

## Abstract

Hematoxylin and Eosin (HE) staining is the gold standard in histopathological examination of cancer tissue, representing the first step towards cancer diagnosis. The second step is a series of immunohistochemical stainings, including cell proliferation markers called the Ki67 index. Deep learning models offer promising solutions for improving medical diagnostics, while generative models provide additional explainability of predictive models that is essential for their adoption in clinical practice. Our previous work introduced a novel approach that utilises a conditional StyleGAN model for generating HE-stained images conditioned on the Ki67 index. This study proposes to employ this model for generating sequences of HE-stained images reflecting varying Ki67 index values. Sequences enable exploration of hidden relationships between HE and Ki67 staining and can enhance the explainability of predictive models, e.g., by generating counterfactual examples. While our previous research focused on assessing the quality of generated HE images, this study extends that work by evaluating the model’s ability to capture Ki67-related variations in HE-stained images. Additionally, expert pathologists evaluated generated sequences and proposed criteria for assessing their relevance. Our findings demonstrate the potential of the conditional StyleGAN model as part of an explainable framework for analysing and predicting immunohistochemical information from HE-stained images. Results highlight the relevance of generative models in histopathology and their potential applications in cancer progression analysis.

## 1. Introduction

Histopathology involves the examination of tissue sections stained by different methods using light microscopy to identify signs of disease [1]. Histopathological analysis represents a condition sine qua non in cancer diagnosis, tumour exact classification, and subsequent treatment recommendations. Tissue staining is a technique that applies one or more dyes to tissue sections in order to enhance contrast and help pathologists identify different diagnostically relevant morphological features of the examined cells. Hematoxylin and Eosin (HE) staining is a gold standard in tissue cancer diagnosis which highlights different microscopically evaluable pathological changes of cancer cells different from those of normal cells in a stained tissue section. Immunohistochemical (IHC) stainings are used to specify the type of cancer by employing antibodies to detect lineage and/or differentiation and prognostic-specific antigens (markers) within cancer tissue sections. In addition, the Ki67 antigen serves as a marker of cell proliferation for assessing Ki67-positive cells that are abnormally growing and dividing, thereby possibly indicating the rate of tumour growth. The Ki67 index quantifies the expression of Ki67-positive cells by calculating the percentage of positive cancer cells in a tissue section. Evaluation of the Ki67 index represents a mandatory step in the cancer diagnostic algorithm; however, IHC Ki67 staining has high demands in terms of resources and time, and its evaluation is more time-consuming than that of HE staining.

Deep learning models show promise for improving medical diagnostics. They can provide fast, consistent, and cost-effective decision-making. However, errors in medical contexts can lead to serious consequences. Therefore, these models must be made explainable prior to implementation in clinical practice in order to prevent potential failures and reveal biases. Model explainability provides transparency, fairness, accuracy, generality, and comprehensibility of the results [2]. Furthermore, the General Data Protection Regulation (GDPR) [3] issued by the European Union requires algorithms to be transparent before being used in patient care.

Integrating a generative model into a deep learning framework can improve explainability by providing additional information to support model predictions. For instance, generative models can produce counterfactual examples derived from minimal modifications to the original data. These result in a change in the model’s prediction, such as a label shift from healthy to unhealthy in the context of medical image analysis. According to [4], pathologists consider counterfactual examples as offering adequate insight into the algorithm’s decision-making criteria. Furthermore, generative models can improve deep learning model performance by augmenting training data [5].

In our previous works [6,7], we proposed a conditional StyleGAN model for generating synthetic HE-stained images conditioned on the Ki67 index, demonstrating promising results in producing realistic synthetic image patches. However, these works primarily focused on the quality of the generated HE images without addressing the Ki67 information obtained from them. In this paper, we extend our analysis by evaluating the Ki67 information within synthetic HE images, aiming to use our model to modify HE images to reflect varying Ki67 index values. Furthermore, we generate sequences of HE-stained images with progressively changing Ki67 index values in order to analyse the relationship between HE and Ki67 staining.

Our research aims to develop an explainable framework for predicting the Ki67 index from HE-stained histological images, with explainability achieved through the use of generative models. The conditional generative model from this paper can be further employed in this explainable framework to explain the Ki67 index predictions from HE-stained images by generating counterfactual examples and simulating cancer progression. Additionally, it can provide deeper insights into the relationship between HE and Ki67 stainings.

The rest of this paper is organised as follows: in Section 2, we discuss generative models and related works; Section 3, describes the dataset, conditional generative model, and evaluation metrics; Section 4 evaluates the model and its latent space, then analyses Ki67 information in HE-stained images; finally, Section 5 presents conclusions and outlines potential directions for future research.

## 2. Background

### 2.1. Generative Adversarial Networks

Generative Adversarial Networks (GANs) were introduced by Goodfellow et al. [8] based on the principle of a min–max optimisation game involving two competing models: a generator *G* and discriminator *D*. The generator aims to learn the distribution of real data and subsequently generate new synthetic samples from this learned distribution that closely resemble the real data. In contrast, the discriminator is trained to distinguish between real data from the real dataset and synthetic data produced by the generator. Both models are trained concurrently and improve simultaneously; as the generator produces more realistic samples, the discriminator improves its ability to accurately differentiate between real and generated data.

The training dataset represents a high-dimensional probability distribution of real data that the generator learns to approximate without explicit specification; thus, it learns this distribution directly from the data samples. As specified by Goodfellow et al. [8], the generator is trained to map G(z), a random latent vector z drawn from a prior distribution $p_{z}$ (typically Gaussian or uniform), into a complex distribution $p_{data}$ approximating real data. The generator is typically implemented as a neural network or convolutional neural network for image data, taking a latent vector as input and outputting a synthetic data sample. In contrast, the discriminator is typically designed as a classification neural network or convolutional neural network that assesses the credibility of input samples, providing a probability score indicating the likelihood that a given sample is real.

GAN models are capable of generating a large number of realistic data samples that closely resemble the training data. However, they are often associated with several problems, including issues with convergence or vanishing gradients. Another common issue is mode collapse, in which the generator produces only a limited subset of the data distribution, manifesting as a lack of diversity in the generated output [9].

Several variations of the original GAN model have been proposed. Conditional GAN models incorporate additional information into both the generator and the discriminator. This enables the model to generate data conditioned on a specific class label, providing more control over the generation process. The conditional GAN concept was introduced in the original GAN paper by Goodfellow et al. [8]. However, it was explored in more detail in the subsequent paper by Mirza et al. [10].

StyleGAN is another improved version of the GAN model, first introduced by Karras et al. [11]. It improves image quality and the structure of latent space to automatically disentangle high-level features, resulting in a more interpretable latent space and providing higher control over the image generation process. StyleGAN involves enhanced generator and discriminator architectures. The generator comprises two networks, a mapping network and a synthesis network. First, the nonlinear mapping network maps latent vectors from the input latent space *Z* to the intermediate space *W*. This intermediate space is disentangled and is not shape-limited [11], allowing the model to capture more meaningful feature representations. Second, the progressively growing synthesis network generates high-resolution images with reduced training time.

### 2.2. Related Work

Several studies have employed generative models in the histopathology domain. Daroach et al. [12] utilised StyleGAN to generate high-resolution 1024×1024 synthetic HE-stained images of prostatic tissue. Leveraging StyleGAN’s progressive growth, this model effectively captures histopathological features across various magnifications. The generated images were deemed to be nearly indistinguishable from real samples by expert pathologists. In their style mixing experiments, Daroach et al. observed that StyleGAN’s hierarchical architecture allows for specific control over image features; coarse layers determine large structures (gland and lumen locations), middle layers define epithelium and stroma textures, and fine layers adjust colour and nuclear density. Furthermore, they explored StyleGAN’s latent space through several experiments, determining representatives for eight histopathological classes by averaging the latent vectors of sample generated images assigned to the same class. These representatives were proven to successfully mimic real histopathological features. Further experimentation as conducted using interpolation between images with different labels. Although the shortest path in latent space yielded realistic images, this did not align with natural biochemical tissue changes. Nevertheless, Daroach et al. demonstrated that the StyleGAN model can generate high-resolution histological images.

In related work, Daroach et al. [13] demonstrated that the latent space of a StyleGAN model trained on a prostate histology dataset of 256×256 patches can capture pathologically significant semantics without data annotation. In that study, they generated a sample of synthetic images with known latent vectors which were subsequently annotated by pathologists. Using these annotations, they identified distinct Gleason pattern-related regions within the latent space by applying principal component analysis. This allowed for the generation of new synthetic images from these regions while preserving the diagnostic features of each region. Pathologists classified images generated from these regions, with 77% of images aligning with the Gleason grade of their region and 98% matching either the same or adjacent diagnostic region. The results indicated that StyleGAN can successfully disentangle prostate cancer features consistently with Gleason grading even without annotated training data. However, the authors emphasised that a balanced training dataset significantly improves the latent space quality.

Quiros et al. [14] introduced PathologyGAN, a model capable of generating 224×224 patches of HE-stained histological images validated by expert pathologists. PathologyGAN is built on the BigGAN architecture [15] with a few modifications. It incorporates a relativistic average discriminator [16] and several StyleGAN [11] features, including intermediate latent space, style mixing regularisation, and adaptive instance normalisation. PathologyGAN was trained on colorectal and breast cancer datasets. The authors verified its construction of an interpretable latent space that captures relevant tissue characteristics and allows for transformation of semantic tissue features through linear vector operations. Additionally, linear interpolation between benign and malignant tissues showed a realistic growing number of cancer cells rather than fading. In [17], Quiros et al. extended their previous work by incorporating an encoder to map real images into latent space, allowinf for analysis of feature representations in a subsequent paper [18].

Schutte et al. [19] proposed the interpretability method to explain black-box model predictions by generating sequences of synthetic images, illustrating the progression of pathology. Their method consists of three parts: StyleGAN to generate synthetic images, an encoder to retrieve a latent representation of generated images, and a logistic regression classifier to approximate the original model’s predictions for generated images. A sequence is generated by traversing the shortest path in StyleGAN latent space, which results in a different model prediction. This approach allows for observation of the changes that impact original model predictions, potentially uncovering new biomarkers [19]. When tested on knee X-rays, their method produced realistic X-ray sequences following osteoarthritis progression. Considering a dataset of breast cancer HE-stained patches, the model generated realistic tumour progression; however, the encoder could not perfectly reconstruct the original histological images.

The studies described above have focused primarily on HE staining, without considering IHC. One promising approach is virtual staining, which transforms HE-stained images into IHC-stained images, enabling IHC analysis without the need for physically stained tissue sections. In this context, Chen et al. [20] introduced the Pathological Semantics-Preserving Learning method for Virtual Staining (PSPStain), specifically designed to preserve essential pathological semantics during the transformation process. Experimental results indicated that PSPStain outperforms existing HE-to-IHC virtual staining methods.

Our previous research [6,7] focused on generating HE-stained tissue images conditioned on the Ki67 index using the conditional StyleGAN model. In [7], we evaluated the training progress and synthetic image quality of several models, analysing the effects of two critical factors: the quality of the training dataset, and the training duration. First, we compared results across two datasets: one containing only high-quality HE-stained patches with sufficient visible cells, and a second comprising a broader range of patches including lower-quality patches and those with fewer visible cells. Afterwards, we compared different training durations to assess their impact on model performance. Additionally, generated images were reviewed and evaluated by the expert pathologist.

While our previous paper deeply analysed the quality of generated images, evaluating Ki67 information hidden in HE images is more challenging. In this paper, we complete the evaluation of this conditional model, incorporating the analysis of latent space and the evaluation of the conditional generator from different aspects. Moreover, we generate sequences of HE-stained images with changing values of the Ki67 index and analyse the relationship between HE and Ki67 staining.

Several of the approaches discussed above address the simulation of cancer progression in histological images. Daroach et al. [12] and Quiros et al. [14] performed interpolation within the latent space between latent vectors of two different images, while Schutte et al. [19] adopted a logistic regression classifier to identify the direction in latent space in order to change the image labels. In contrast, our approach modifies the original image by adjusting the conditioning input for the conditional generator.

## 3. Methods

We use the conditional StyleGAN model presented in [7], where we provided a detailed analysis of image generation quality and validation by the pathologist. In that work, the expert pathologist confirmed the quality of the synthetic images, describing them as highly realistic. Synthetic images could only be distinguished from real ones upon close and detailed examination. However, in that work we omitted analysis of the Ki67 information within generated HE-stained images as well as analysis of the latent space. In this paper we conduct a detailed evaluation of these aspects.

### 3.1. Dataset

We employ the dataset presented in our previous paper [21]. The unprocessed histopathological dataset was provided by the Department of Pathology, Jessenius Medical Faculty of Comenius University and University Hospital Martin. It consists of HE- and Ki67-stained whole-slide images of seminoma, a testicular tumour. This dataset comprises 77 pairs of HE-stained and Ki67-stained digital tissue scans, with each pair created from adjacent tissue sections. Although the sections do not match exactly at the cellular level, we assume that tissue sections from the same region share similar characteristics. Consequently, we utilise Ki67-stained images to annotate the adjacent HE-stained images.

**Figure 1:**
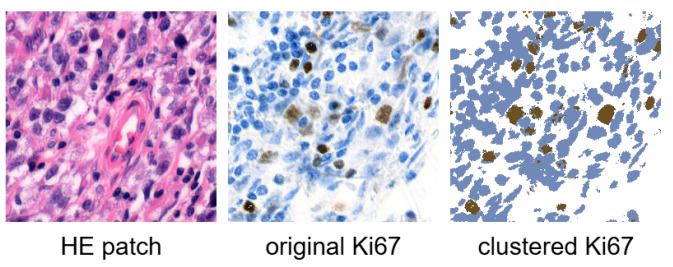
Example of corresponding HE patch, original Ki67 patch, and clustered Ki67 patch. The Ki67 index is equal to 0.1 [6].

In this paper, we use the processed dataset initially introduced in our previous work [22] and subsequently employed to train the conditional StyleGAN model in [6,7]. The processed dataset was prepared through the semi-automated annotation approach, as the unprocessed dataset lacked annotations. This process involved three main steps: tissue registration, colour-based clustering, and Ki67 index quantification. Due to the large size of whole-slide images and computational constraints, the images were divided into smaller 256×256 square patches at the second-highest resolution level. Corresponding HE and Ki67 patches were cut from the same positions. Each HE patch was subsequently labelled with the Ki67 index, which was computed as the proportion of positively stained (brown) pixels in the corresponding clustered Ki67 patch. Figure 1 illustrates an example of an HE patch with its corresponding Ki67 patch, including the Ki67 patch after clustering. The Ki67 index of this particular patch is 0.1 (10%).

**Figure 2:**
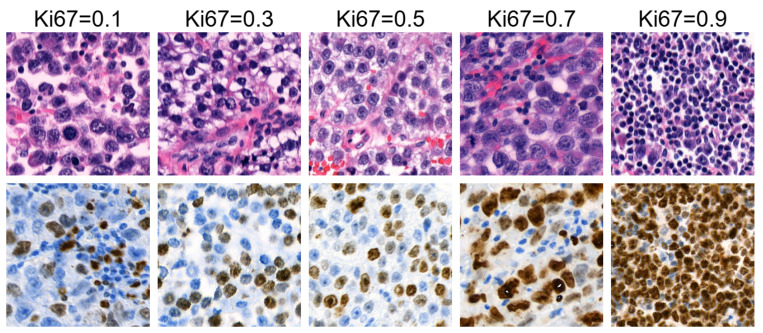
Examples of HE patches with Ki67 index labels from the filtered dataset alongside their corresponding Ki67 patches.

**Figure 3:**
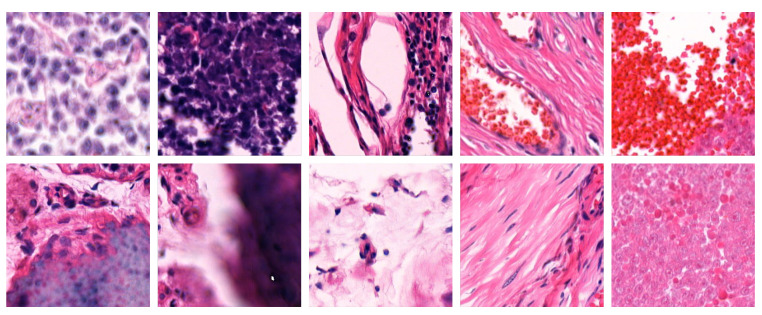
Examples of HE patches excluded by our filter [7].

To ensure the quality and relevance of the patches, we applied a comprehensive filtering process, the details of which are presented elsewhere. The filtering involved edge detection, blur detection, blob detection, and clustering techniques to exclude patches with lower quality or insufficient cellular content. As a result, the filtered dataset consists of 177,907 patches derived from 77 tissue scans, with each HE-stained patch labelled with its corresponding Ki67 index. Figure 2 illustrates examples of HE patches included in the filtered dataset along with their corresponding Ki67 patches and Ki67 index labels, while Figure 3 shows example patches that were excluded by the filtering process.

### 3.2. Generative Model

**Figure 4:**
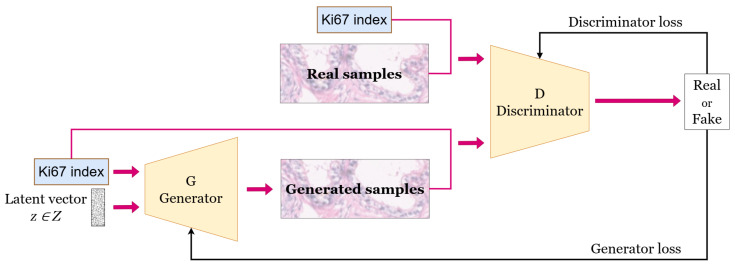
Conditional GAN [7].

We utilised the generative model introduced in our earlier work [7], which is a conditional StyleGAN model that generates HE-stained image patches conditioned on the Ki67 index. The architecture of the conditional generative model is illustrated in Figure 4. It takes a latent vector z∈Z as input and uses a specified Ki67 index to condition the generation of the HE-stained patches.

**Figure 5:**
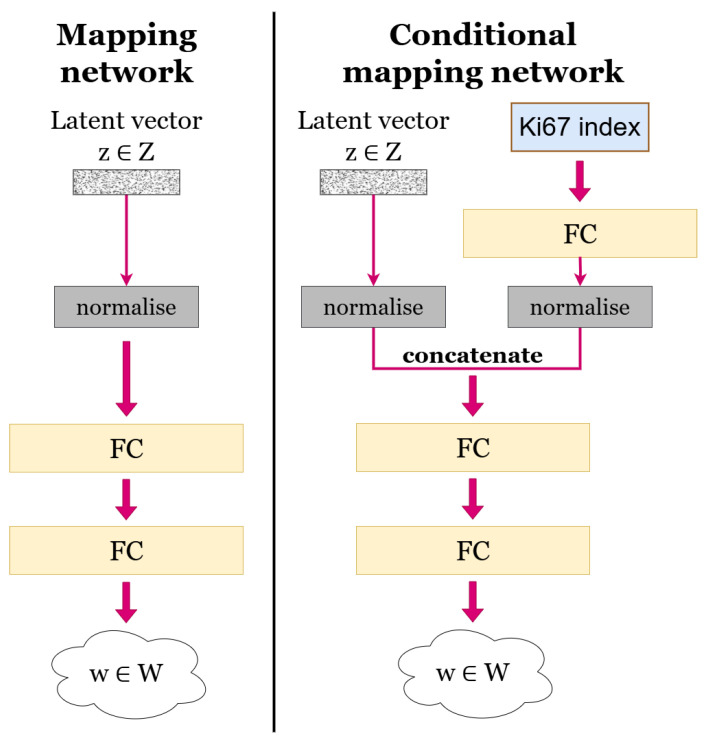
Comparison of unconditional and conditional mapping networks.

Conditioning is applied within the StyleGAN mapping network following the official implementation by NVlabs [23]. Figure 5 compares the traditional unconditional mapping network (left) with the conditional version (right). In the conditional mapping network, the Ki67 index is first passed through a fully connected (FC) layer to produce a vector with the dimension of the intermediate latent space *W*. This vector is then normalised in the same way as the input latent vector *z*. The two vectors are concatenated and passed through two FC layers, as in the original mapping network, resulting in the final intermediate latent vector w∈W.

Daroach et al. [12] successfully applied StyleGAN [11] and StyleGAN2 [24] for histopathology image synthesis, which motivated our choice of the improved alias-free successor, StyleGAN3, proposed by Karras et al. [23]. Specifically, we adopt the StyleGAN3-R variant; this architecture maintains equivariance to both translation and rotation, which is convenient for histological images.

The model was trained using the official implementation from NVlabs with the regularisation parameter gamma set to 2 and adaptive discriminator augmentation enabled. Training was conducted on the filtered dataset, which consists of pairs of HE image patches and corresponding Ki67 index values. For most experiments, we utilised the model trained for 5343 kimgs, indicating that the model processed 5,343,000 HE patches during the training phase; thus, it iterated through the whole dataset approximately 28 times. In a few experiments, we also employed the model trained for 10,000 kimgs in order to compare its performance with that of the 5343 kimgs model.

### 3.3. Evaluation Metrics

We use three metrics—Fréchet Inception Distance, Fréchet Histological Distance, and Perceptual Path Length—to evaluate the generative model’s performance, considering the quality of the generated images and the latent space. All these metrics are minimised for more realistic generated images and improved latent space quality. Together, these metrics provide a comprehensive evaluation of synthetic histological images by assessing visual and histological fidelity, diversity, and latent space disentanglement.

The Fréchet Inception Distance (FID) [25] offers a quantitative assessment of how closely generated images approximate real-world data in terms of quality and diversity. This metric evaluates images based on high-level feature representations [26] from a pretrained inception model, which aligns with human judgment. Specifically, the FID measures the distance between the distributions of generated and real images by calculating the Fréchet distance [27] between two Gaussian distributions.

The second metric is the Fréchet Histological Distance (FHD), which is a modified version of the FID designed specifically for histological images. Because the inception model is trained on ImageNet, it does not consider histological features. To address this, we replaced the inception model with a histological model in which images are represented as high-level features of the histological model [22]. This model was then trained to predict the Ki67 index from the HE-stained images.

The Perceptual Path Length (PPL) was introduced along with the StyleGAN model [11] to evaluate the quality of latent space. It assesses the entanglement of latent space by quantifying the perceptual smoothness of interpolation between latent vectors. The perceptual distance between two images is calculated using VGG16 [28] embeddings.

## 4. Results

This section evaluates the conditional generator and its latent space, with a focus on the Ki67 index. We propose sequences of HE-stained images generated by modifying the Ki67 index, then analyse the relationship between the generated HE images and their corresponding Ki67 index values.

### 4.1. Analysis of Training Progress

**Figure 6:**
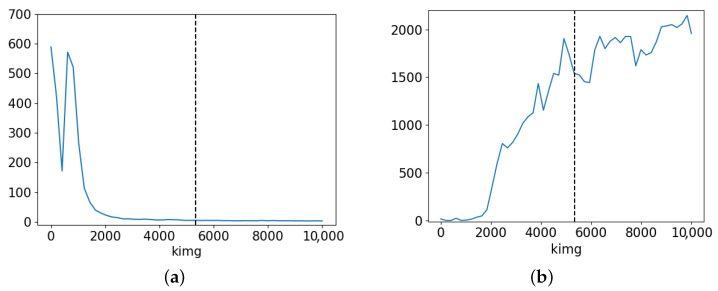
Training progress in terms of FID and PPL metrics [7]: (**a**) FID scores and (**b**) PPL scores.

In our previous paper [7], we demonstrated the proposed model’s superior results on a high-quality filtered dataset, particularly in terms of image quality and training stability. Figure 6 illustrates the FID and PPL progression during the training of this model. It is evident that while FID decreases as expected, the PPL shows an increasing trend. This increase in PPL is likely due to the model overfitting to the data; hence, the distance between the real and synthetic data distribution decreases, but the latent space grows more entangled. This phenomenon may be linked to mode collapse, in which the model generates a limited variety of images.

While Figure 6 suggests that the model trained for 2000 kimgs achieved low values for both FID and PPL, visual inspection revealed that the generated images were less realistic. Expert pathologists evaluated images as realistic only after 5000 kimgs of training. Consequently, we selected the model with a training duration of 5343 kimgs for most of our subsequent experiments, as indicated by the vertical line in the figure. This choice represents a compromise between FID and PPL, achieving a balance between image quality and latent space structure. Specifically, the selected conditional StyleGAN3-R model achieved an FID of 5.5, FHD of 10.52, and PPL of 1541.35. In comparison, the unconditional StyleGAN3-R variant produced lower image quality, with an FID of 14.79 and FHD of 42.81, while significantly outperforming it in terms of latent space structure, achieving a PPL of 24.64. The conditional StyleGAN3-T model achieved performance results that were between these two models in terms of image quality metrics (FID 9.39, FHD 17.45), but exhibited the weakest latent structure, with a PPL of 85333.9. All FID and FHD scores were computed by comparing synthetic images generated by the corresponding model with real HE-stained images from our dataset.

### 4.2. Evaluation of the Conditional Generator

A conditional generator produces data with a specified property. In our case, it generates HE-stained histological images conditioned on a given Ki67 index. To assess whether the generated images effectively incorporate the Ki67 information, we compared real HE-stained images from our dataset with synthetic images generated across various Ki67 index intervals. This comparison provides a quantitative validation of the presence and fidelity of Ki67-related information in the generated HE-stained images.

**Figure 7:**
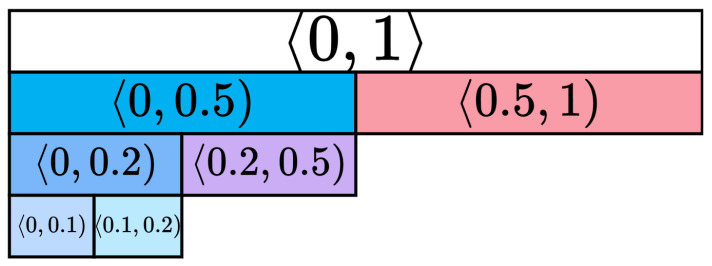
Ki67 intervals.

Ki67 index values are within the 〈0,1〉 range. To evaluate the generator’s ability to generate HE-stained images with the specific Ki67 indexes, we divided the full Ki67 range into smaller Ki67 intervals, which were chosen as follows: 〈0.5,1〉, 〈0,0.5), 〈0.2,0.5), 〈0,0.2), 〈0.1,0.2), 〈0,0.1). The use of Ki67 index intervals for seminoma tumours is not standardised, and there is no consensus regarding the optimal cut-off values; therefore, intervals were defined based on pathologists’ recommendations and considering the dataset distribution over Ki67 index labels. Certain intervals are hierarchical, with one interval being a subset of another, while others are complementary, thereby representing non-overlapping datasets. The hierarchy of intervals is illustrated in Figure 7.

**Figure 8:**
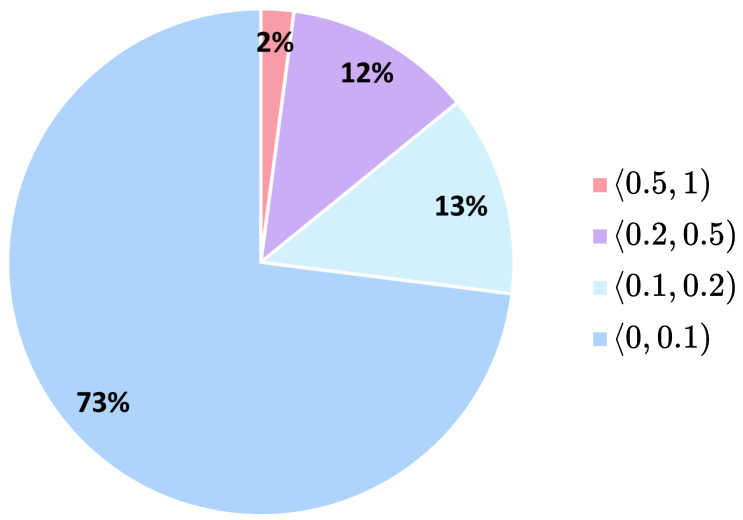
Distribution of real HE-stained images across Ki67 intervals in the dataset.

Before evaluating the generator, we assigned the real HE-stained images from our dataset to defined Ki67 intervals according to their Ki67 index labels. The distribution of real data across Ki67 intervals in the dataset is shown in Figure 8. It can be observed that the Ki67 index labels are highly imbalanced, with the majority of the data labelled with a Ki67 index below 0.1 and only 2% of the data having a Ki67 index above 0.5. Second, we created synthetically generated Ki67 intervals by randomly generating synthetic HE images for each Ki67 interval using our conditional generator. For each evaluation, we generated 50,000 new synthetic HE images. Finally, we analysed the relationships between the dataset and the generated Ki67 intervals to assess the generator’s conditional performance.

#### 4.2.1. Evaluation Using Fréchet Inception Distance

In our first evaluation of the generator’s performance, we scrutinised the visual similarity between Ki67 intervals. For this purpose, we adopted the Fréchet Inception Distance score, which measures the difference in the feature distribution between real data and generated data using the inception model. FID scores were calculated between all of the dataset’s Ki67 intervals of real images and all of the generated Ki67 intervals of synthetic images produced by the conditional generator. The results are presented in Table 1, where rows correspond to dataset intervals and columns represent generator intervals; hence, the values in the Table are not symmetric. We highlight the hierarchical structure of the intervals in the row and column descriptions through different colours.

Each value in the table represents the FID score calculated between images from the corresponding real and generated intervals, reflecting the distance between their feature distributions. Lower FID scores indicate more similar distributions, suggesting that images from the generated interval more closely resemble those from the dataset interval. Lower FID scores are expected between subset intervals, and are highlighted in the table with a green background. Conversely, higher FID scores are expected for complementary intervals, and are distinguished with a yellow background. Unexpected FID scores are emphasised with red-coloured text. The lowest FID scores are anticipated along the table’s diagonal, where the real and generated intervals are identical. These diagonal FID scores are marked in bold. Appendix A includes Table A1, which complements Table 1 by presenting FID scores with a heatmap background for a more intuitive visualisation of FID score differences.

From analysing the results presented in Table 1, it is evident that the interval 〈0.5,1〉 is complementary to all other intervals. As expected, the lowest FID score is found along the diagonal. When comparing the dataset interval 〈0.5,1〉 with all generator intervals in the row, the interval 〈0.2,0.5) exhibits the closest FID score to the diagonal, corresponding to the closest interval in terms of Ki67 distribution.

Next, we analyse the interval 〈0,0.5). As anticipated, the FID score with the complementary interval 〈0.5,1〉 is high. The remaining intervals are subsets of 〈0,0.5); as expected, the FID scores are lower compared to the complementary interval 〈0.5,1〉. The highest FID score is associated with the interval 〈0.2,0.5), but is still more than two times lower than the score for the complementary interval 〈0.5,1〉. This is due to the fact that the majority of the real dataset within the interval 〈0,0.5) consists of images with low Ki67 index values.

The Ki67 interval 〈0.2,0.5) exhibits the least favourable performance in the table. For the 〈0.2,0.5) generator interval in the column, the FID score with the complementary dataset interval 〈0.1,0.2) is lower than the diagonal score, indicating that the generated images are more similar to real images from the 〈0.1,0.2) interval than to those from the 〈0.2,0.5) interval. Furthermore, the 〈0.2,0.5) dataset interval in the row shows a shorter distance to the complementary intervals 〈0,0.2), 〈0.1,0.2), and 〈0,0.1). This can be attributed to the imbalanced distribution of the real data. However, the interval shows the expected high FID score with the complementary 〈0.5,1〉 interval and the expected low score with 〈0,0.5).

The remaining intervals—〈0,0.2), 〈0.1,0.2), and 〈0,0.1)—all have expected FID scores, with lower values for subset intervals and higher values for complementary intervals. Along with 〈0,0.5), these intervals exhibit the lowest diagonal FID scores, as they contain the majority of real data; in contrast, the interval 〈0.5,1〉 has the highest diagonal FID, as it contains the smallest number of real HE images. Therefore, the conditional generator demonstrates the highest performance in terms of visual similarity for the lower Ki67 index values.

#### 4.2.2. Evaluation Using Fréchet Histological Distance

In addition to visual similarity, we evaluated the histological similarity between Ki67 intervals using the Fréchet Histological Distance. FHD quantifies the distance between the feature distributions of real and generated HE images using the histological model.

FHD results for the dataset vs. generator Ki67 intervals are summarised in Table 2, which follows the same structure as described in Section 4.2.1. Additionally, Table A2 in Appendix A presents FHD values with a heatmap background for enhanced visual representation. The FHD results are proportionally similar to the FID scores in Table 1, with a slight improvement. Although the generator interval 〈0.2,0.5) in the column again shows a lower distance to the dataset interval 〈0.1,0.2) than the diagonal FHD score, the dataset interval 〈0.2,0.5) in the row has the lowest FHD score along the diagonal, as expected. However, the diagonal FHD for the interval 〈0.5,1〉 remains significantly higher than the diagonal FHD of other intervals.

Subsequently, we evaluated the generator vs. generator FHD scores to verify that the generator captures relevant histological characteristics for specific Ki67 intervals. The results are presented in Table 3 and by the heatmap in Table A4 of Appendix A. These tables follow the same structure as the previous tables in this section. However, in this case both the rows and columns represent generator intervals, making the table symmetric. The results are analogous to the dataset vs. generator results, where the interval 〈0.2,0.5) shows a smaller distance to the complementary interval 〈0.1,0.2) than to 〈0,0.5). Apart from this, all other results align with expectations.

Additionally, we evaluated FHD scores for the dataset vs. dataset Ki67 intervals in order to verify the properties of our dataset. The results are summarised in Table 4 and by the heatmap in Table A3 of Appendix A. The tables have a similar structure to the generator vs. generator tables, but both the rows and columns correspond to dataset intervals, making this table symmetric as well. The results reflect the Ki67 imbalance in the dataset, with the most accurate results observed for the lower Ki67 indices. The only notable inconsistency is that the Ki67 interval 〈0.2,0.5) appears more similar to the complementary interval 〈0.1,0.2) than to 〈0,0.5), which is a consequence of the majority of data in the 〈0,0.5) interval having Ki67 labels close to 0. This inconsistency is also reflected in the behaviour of our conditional generator, and as such can be observed in all of the previously presented tables.

#### 4.2.3. Evaluation Using Perceptual Path Length

In addition to visual and histological similarity, we analysed the conditional properties of the latent space using Ki67 intervals and the Perceptual Path Length (PPL), which measures latent space entanglement. The corresponding results are presented in Table 5. The PPL score for the entire Ki67 range 〈0,1〉 is 1526.11, which is close to the average score across all evaluated Ki67 intervals. Notably, the PPL scores exhibit minimal variation, leading to the conclusion that the disentanglement of the latent space is consistent across all Ki67 intervals.

#### 4.2.4. Discussion

The results presented in these tables demonstrate adequate relationships between various Ki67 intervals as validated by FID and FHD scores. However, slightly worse performance is observed for the interval 〈0.2,0.5) in all the FID and FHD tables, and a higher diagonal score is noted for the interval 〈0.5,1〉 in the dataset vs. generator evaluations. These discrepant results can be attributed to the highly imbalanced dataset, in which a majority of the data exhibits low Ki67 indices. Nevertheless, the findings in the tables confirm the generator’s ability to conditionally generate HE-stained images that correspond to the specified Ki67 index. Additionally, the PPL results validate the consistent disentanglement of the latent space across Ki67 conditions.

### 4.3. Analysis of Ki67 Expression in HE-Stained Images

To analyse the Ki67 expression within HE-stained images, we generated sequences of HE images that correspond to varying Ki67 index values. This approach allowed us to scrutinise how variations in the Ki67 index are reflected in HE-stained images, providing insights into potential correlations between HE staining patterns and Ki67 expression. Due to the insufficient number of images in the dataset with Ki67 indices above 0.5, we focused this analysis exclusively on Ki67 index values ranging from 0 to 0.5 in order to ensure reliable results. Furthermore, sequences with Ki67 indices above 0.5 were evaluated by pathologists as being less realistic.

The sequences were generated according to the following rules:

- Each sequence contained six images.
- Images within each sequence were generated from the same randomly selected input latent vector, while the input latent vector differed between sequences.
- The Ki67 index started at Ki67 = 0 for the first image and increased to Ki67 = 0.5, with a step size of 0.1.

We generated two groups of sequences, each containing 20 sequences; the first group was generated using the conditional StyleGAN model trained for 5343 kimgs, while the second group was generated using the model trained for 10,000 kimgs. Both groups of sequences were evaluated by two expert pathologists.

#### 4.3.1. Analysis of the First Group of Sequences

**Figure 9:**
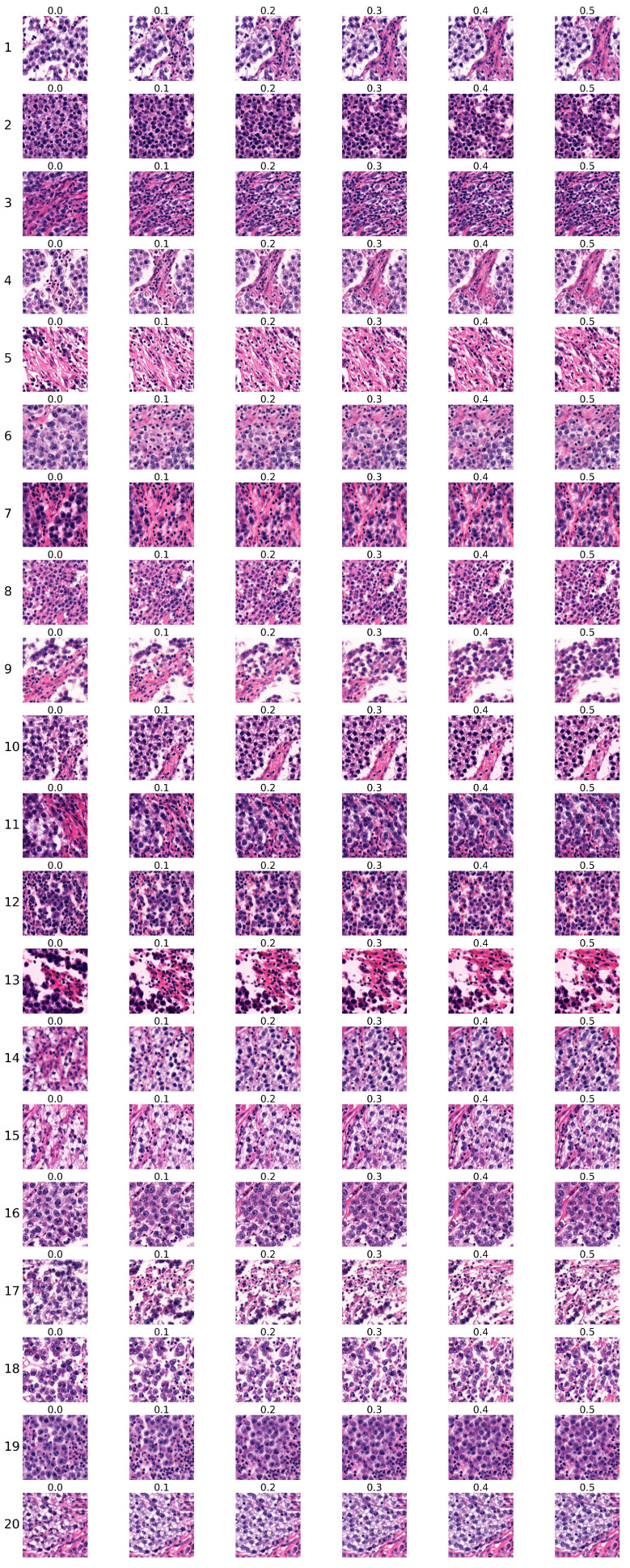
The first group of sequences generated by the conditional StyleGAN model trained for 5343 kimgs.

The first group of sequences was generated using the conditional StyleGAN model trained for 5343 kimgs. Sequences are shown in Figure 9. Each row corresponds to one sequence, with the sequence number indicated on the left, while each column corresponds to the specific Ki67 index, which is denoted above each image.

Two expert pathologists independently evaluated each sequence, and their assessments are presented in Table 6. Each pathologist marked each sequence as `certainly real’, `rather real’, `certainly unreal’, `rather unreal’, or `partially real and unreal’. The first pathologist’s choices are marked by `1’ and the second pathologist’s choices by `2’, with different colours used to visually distinguish their selections.

Before evaluating the sequences, we analysed the consistency of the pathologists’ assessments. The distances between their evaluations are summarised in Table 7. If we consider an evaluation to be inconsistent when the distance exceeds one, we find that 70% of the pathologists’ assessments are consistent, with the maximum observed distance being 2.

The pathologists’ evaluation of the generated sequences is summarised in Table 8. The first pathologist rated 45% of the sequences as real, 40% as partially real, and 15% as unreal. Similarly, the second pathologist evaluated 45% of the sequences as realistic, 20% as partially real, and 35% as unreal. Together, the pathologists assessed 45% of sequences as real, 30% as partially real, and 25% as unreal. These results indicate that the majority of generated sequences were perceived as either realistic or partially realistic, suggesting that they plausibly reflect actual biological processes. According to both pathologists, the most realistic sequences were 3, 7, 11, 15, and 16.

The pathologists concluded that the sequences generated by the model trained on whole-slide images of seminoma samples reproduced the tumour histopathology in a highly realistic manner, including the typical cytology of tumour cells and inflammatory immune response. The generated images copied the tissue arrangement corresponding to real tissue with no apparent central or axial symmetry artifacts, to the degree where they were practically indistinguishable from real microphotographs.

Both pathologists independently established their evaluation criteria for rating the Ki67 sequences. Changes such as increasing percentage and/or density of tumour cells, decreasing amount of supportive non-tumour tissue (the so-called stroma), arrangement corresponding to real tissue, and increase of inflammatory cells were evaluated as ’certainly real’ or ’rather real’ by one of the pathologists.

One pathologist considered findings where the tumour population exhibited changes but did not grow in number as less realistic due to the failure of the sequence to produce more tumour cells. Another pathologist evaluated as more real those sequences displaying the same number of tumour cells but showing cytological changes such as increased density of nuclear chromatin or increased eosinophilia of cytoplasm, potentially indicating markers of more rapid growth even in real tissue.

Changes that appeared to be inconsistent such as a fluctuating number of tumour and inflammatory cells, were evaluated as ’partially real and unreal’. The morphology was consistent with real images; however, the differences between the sequences were minimal, difficult to compare, or apparently random, making it impossible to determine the pattern of change. This criterion was consistent among both pathologists.

Changes where the model performed poorly were evaluated as `certainly unreal’ or `rather unreal’; these included areas of nonvital tissue (so-called necrosis) increasingly present in sequences with high Ki67 index, which were evaluated as less realistic (Sequence 6 in Figure 9). This type of regressive change does not react with Ki67 antigen due to the lack of preserved nuclei in necrotic areas. The increase of images resembling tumour stroma was also evaluated similarly (Sequence 4 and Sequence 5 in Figure 9). This tissue normally does not display a high proliferation rate; therefore, its presence is expected to decrease with increasing Ki67.

#### 4.3.2. Analysis of the Second Group of Sequences

**Figure 10:**
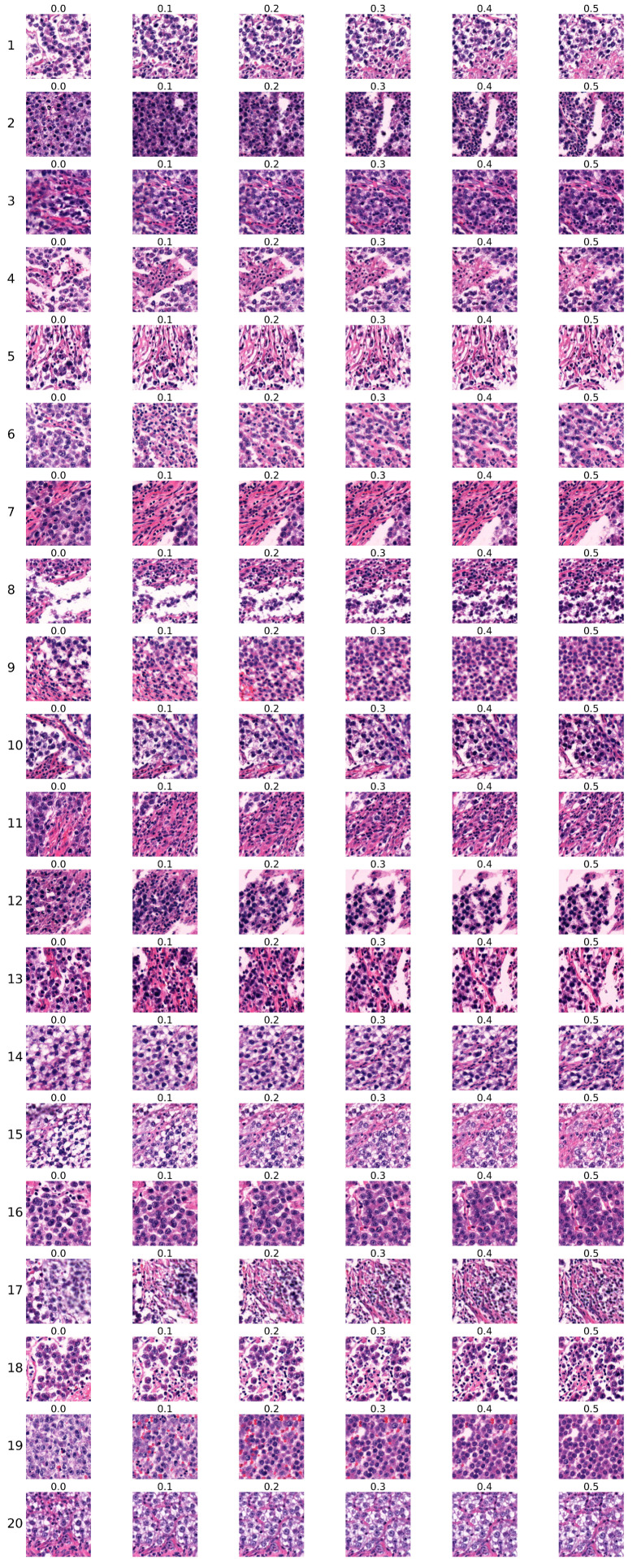
The second group of sequences generated by the conditional StyleGAN model trained for 10,000 kimgs.

The second group of sequences was generated using the model trained for 10,000 kimgs. Sequences are shown in Figure 10. The figure is organised in the same manner as the first group in Figure 9 in the previous section, with each row representing one sequence and each column corresponding to the particular Ki67 index.

Similar to the first group, sequences were evaluated by two expert pathologists. The results are presented in Table 9, which follows the same structure as Table 6 in Section 4.3.1 describing the results for the first group. Initially, we analysed the consistency of the pathologists’ assessments. The distances between their evaluations are presented in Table 10. Defining a distance greater than 1 as inconsistent, we find that 55% of the assessments demonstrate consistency, reflecting a lower level of consistency than observed in the first group of sequences. Furthermore, the Sequence 10 shows a distance of 4 between the pathologists’ assessments.

Table 11 provides a summary of the pathologists’ assessments of the second group of sequences. The pathologists rated this group as being less realistic and less consistent than the first group. The first pathologist classified 50% of sequences as real, 15% as partially real, and 35% as unreal. Similarly, the second pathologist’s evaluation indicated lower realism, with 35% of sequences rated as real, 15% as partially real, and 50% as unreal. Together, their evaluations considered 42.5% of the sequences to be real, 15% to be partially real, and 42.5% to be unreal. The proportions of sequences rated as real and unreal are identical. Therefore, we can conclude that the sequences in the second group were less realistic than those in the first group. According to the assessments of both pathologists, sequences 3, 9, 16, and 18 were the most realistic.

The pathologists’ evaluation was performed using the same criteria as described in the previous section. Sequences evaluated as `certainly real’ or `rather real’ showed a consistent increase of tumour and/or inflammatory cells, a decrease in supportive tissue, and increased density of observed changes. Sequences evaluated as `partially real and unreal’ failed to display one consistent pattern, fluctuating between increase and decrease of tumour cells. The `certainly unreal’ or `rather unreal’ rating was reserved for images containing necrosis in all images of the sequence, generated sections of random non-tumour tissue, and/or an increase in the blank white space corresponding to no tissue or tearing artifacts.

#### 4.3.3. Discussion

The first group of sequences generated by the conditional StyleGAN model trained for 5343 kimgs was evaluated by the pathologists as being more realistic, with 45% of sequences assessed as real and 30% as partially real. The pathologists’ assessments for this group showed a 30% inconsistency rate.

In contrast, the second group of sequences produced by the conditional StyleGAN model trained for 10,000 kimgs was considered less realistic, with 42.5% of sequences rated as unreal. The pathologists also demonstrated lower agreement in their evaluations of this group, with 45% of assessments showing inconsistency.

The largest observed discrepancies were a result of variations in the evaluation criteria. The first pathologist (1) considered a change in tumour cytology to be a real finding even without a consistent increase of tumour cells, while the second pathologist (2) regarded failure to generate a steadily increasing amount of tumour cells as being less realistic.

In summary, the conditional StyleGAN model trained for 5343 kimgs generated sequences with more realistic variations in Ki67 expression within HE-stained images, albeit with lower image quality. In contrast, the model trained for 10,000 kimgs produced higher-quality images but showed less realistic Ki67 index variation. These results align with the observations in Section 4.1, where the PPL was found to increase during training, suggesting that while image quality improved, the quality of the latent space diminished, leading to less realistic Ki67 index variations.

## 5. Conclusions

In this paper, we have extended our prior works [6,7] by scrutinising the generation of HE-stained histological images conditioned on the Ki67 index by utilising a conditional StyleGAN model. Our previous studies evaluated the quality of generated HE-stained images without addressing the Ki67 information. Hence, this paper focuses on the model’s ability to capture Ki67 expression in HE-stained images.

Our analysis of the model’s conditional performance in generating HE-stained images confirms its alignment with Ki67 index conditioning. Using the FID and FHD metrics, we validated the correspondence of Ki67 intervals between generated and real data. However, slightly lower performance was noted in the intervals 〈0.2,0.5) and 〈0.5,1〉 due to dataset imbalance, where most of the data exhibit lower Ki67 indices. Nevertheless, these results confirm our model’s reliability in generating HE-stained images conditioned on the Ki67 index. Furthermore, the PPL scores demonstrate consistent latent space disentanglement across all Ki67 intervals. Our future work will explore techniques for handling imbalanced datasets, e.g., data augmentation or resampling. Incorporating these strategies in future research could further improve the reliability of conditional generation.

In the second part of the paper, we generated sequences of HE-stained images with varying Ki67 indices. These sequences enabled us to scrutinise how changes in Ki67 expression are reflected in HE-stained images, offering insights into potential relationships between HE and Ki67 staining patterns. Expert pathologists concluded that the generated sequences accurately replicate the histopathological characteristics of tumours. The experts’ evaluation of the generated sequences leads to the following conclusions. The first group of sequences produced by the model trained for 5343 kimgs was considered more realistic, with 45% of sequences assessed as real and 30% as partially real. This group had a 30% inconsistency rate between the two pathologists’ assessments. In contrast, sequences from the model trained for 10,000 kimgs were deemed less realistic, with 42.5% rated as unreal and 45% disagreement between the pathologists.

Our results demonstrate that the conditional StyleGAN model trained for 5343 kimgs produces sequences with more realistic variations in Ki67 expression, although with lower image quality. In contrast, the model trained for 10,000 kimgs produces higher-quality images, but these less accurately reflect the Ki67 index variation. This outcome aligns with our findings that the FID values decreased during training, while the PPL values increased. This indicates that while visual fidelity improves, the structure of the latent space becomes more entangled, leading to less realistic Ki67 index variations. The results suggest that the model trained for a shorter duration offers a better balance between latent space and image quality. Thus, our findings show a tradeoff in conditional generative modelling for histopathological applications. Future work will explore advanced approaches for increasing the disentanglement in the latent space in order to improve both image and latent space quality, supporting applications of our model in predictive histopathology and cancer progression analysis. One potential research direction is exploring alternative StyleGAN variants. While the currently selected StyleGAN3-R model achieved an FID score of 5.5, FHD of 10.52, and PPL of 1541.35, preliminary experiments with the StyleGAN2-ADA model yielded significantly better results, achieving an FID score of 1.65, FHD of 2.96, and PPL of 12.13.

The conditional StyleGAN model offers a promising foundation for generating synthetic HE-stained images that accurately reflect Ki67 expression. Future directions include leveraging generative models to develop an explainable framework for predicting and analysing the Ki67 index from HE-stained images, with potential applications in counterfactual analysis and cancer progression simulations.

## Figures and Tables

**Table 1:** FID results on dataset vs. generated Ki67 intervals.

			Generator					
			**<0.5,1>**	<0,0.5)				
					**<0.2,0.5)**	**<0,0.2)**		
**Dataset**							**<0.1,0.2)**	**<0,0.1)**
**<0.5,1>**			**15.16**	19.10	16.45	19.85	17.18	20.52
**<0,0.5)**			19.72	**5.67**	9.80	5.52	7.13	5.59
	**<0.2,0.5)**		20.50	8.17	**9.90**	8.42	9.45	8.57
	**<0,0.2)**		22.12	5.45	10.26	**5.59**	7.24	5.65
		**<0.1,0.2)**	19.53	6.52	8.76	6.59	**6.61**	7.01
		**<0,0.1)**	20.77	5.86	10.75	5.67	7.72	**5.64**

**Table 2:** FHD results on the dataset vs. generated Ki67 intervals.

			Generator					
			**<0.5,1>**	<0,0.5)				
					**<0.2,0.5)**	**<0,0.2)**		
**Dataset**							**<0.1,0.2)**	**<0,0.1)**
**<0.5,1>**			**32.41**	43.08	33.39	45.68	37.62	47.95
**<0,0.5)**			45.40	**10.36**	19.85	10.41	12.95	10.83
	**<0.2,0.5)**		35.47	23.42	**20.71**	25.70	22.31	26.67
	**<0,0.2)**		44.97	10.49	21.05	**10.45**	13.70	10.77
		**<0.1,0.2)**	37.01	15.28	17.56	16.51	**13.07**	17.83
		**<0,0.1)**	47.78	11.32	23.86	11.14	15.31	**11.03**

**Table 3:** FHD results for generator vs. generator Ki67 intervals.

			Generator					
			**<0.5,1>**	<0,0.5)				
					**<0.2,0.5)**	**<0,0.2)**		
**Dataset**							**<0.1,0.2)**	**<0,0.1)**
**<0.5,1>**			**0.42**	33.87	13.11	38.12	29.10	41.70
**<0,0.5)**			33.87	**0.45**	9.99	0.70	3.27	1.05
	**<0.2,0.5)**		13.11	9.99	**0.44**	12.92	5.72	15.35
	**<0,0.2)**		38.12	0.70	12.92	**0.44**	4.41	0.61
		**<0.1,0.2)**	29.10	3.27	5.72	4.41	**0.42**	6.00
		**<0,0.1)**	41.70	1.05	15.35	0.61	6.00	**0.44**

**Table 4:** FHD results for the dataset vs. dataset Ki67 intervals.

			Generator					
			**<0.5,1>**	<0,0.5)				
					**<0.2,0.5)**	**<0,0.2)**		
**Dataset**							**<0.1,0.2)**	**<0,0.1)**
**<0.5,1>**			**0.00**	40.95	31.47	44.38	32.75	47.80
**<0,0.5)**			40.95	**0.00**	12.82	0.30	6.19	0.87
	**<0.2,0.5)**		31.47	12.82	**0.00**	16.81	9.43	19.56
	**<0,0.2)**		44.38	0.30	16.81	**0.00**	7.72	0.24
		**<0.1,0.2)**	32.75	6.19	9.43	7.72	**0.00**	10.58
		**<0,0.1)**	47.80	0.87	19.56	0.24	10.58	**0.00**

**Table 5:** PPL results for Ki67 intervals.

<0,1>						
	<0.5,1>	<0,0.5)				
			<0.2,0.5)	<0,0.2)		
					<0.1,0.2)	<0,0.1)
1526.11	1561.95	1549.54	1496.20	1533.52	1482.50	1559.26

**Table 6:** Pathologists’ evaluation of the first group of sequences generated by the conditional StyleGAN model trained for 5343 kimgs.

Sequence Order	Certainly Unreal	Rather Unreal	Partially Real and Unreal	Rather Real	Certainly Real
1			1		2
2			1	2	
3				1 2	
4		1 2			
5			1 2		
6			2	1	
7				1 2	
8	2		1		
9		2		1	
10		2	1		
11				1 2	
12	2		1		
13		1 2			
14		2		1	
15				1	2
16				1	2
17		1		2	
18			1 2		
19			2	1	
20			1	2	

**Table 7:** Distances between pathologists’ evaluations of the first group of sequences.

Distance	Number of Sequences
0	7
1	7
2	6
3	0
4	0

**Table 8:** Summary of pathologists’ evaluation of the first group of sequences.

	Certainly Unreal	Rather Unreal	Partially Real and Unreal	Rather Real	Certainly Real
Pathologist 1	0	3	8	9	0
Pathologist 2	2	5	4	6	3
Pathologist 1 and 2	2	8	12	15	3

**Table 9:** Pathologists’ evaluation of the second group of sequences generated by the conditional StyleGAN model trained for 10,000 kimgs.

Sequence Order	Certainly Unreal	Rather Unreal	Partially Real and Unreal	Rather Real	Certainly Real
1		1 2			
2		1 2			
3				1	2
4	2	1			
5		1 2			
6			1		2
7		1	2		
8		2		1	
9				1 2	
10	2				1
11	2			1	
12		2	1		
13		1			2
14		2		1	
15			1	2	
16				1	2
17	1		2		
18				1	2
19			2		1
20		2		1	

**Table 10:** Distances between pathologists’ evaluations of the second group of sequences.

Distance	Number of Sequences
0	4
1	7
2	6
3	2
4	1

**Table 11:** Summary of pathologists’ evaluations of the second group of sequences.

	Certainly Unreal	Rather Unreal	Partially Real and Unreal	Rather Real	Certainly Real
Pathologist 1	1	6	3	8	2
Pathologist 2	3	7	3	2	5
Pathologist 1 and 2	4	13	6	10	7

## Data Availability

The original data presented in the study are available at https://doi.org/10.5281/zenodo.11218961 (accessed on 1 June 2024).

## Abbreviations

The following abbreviations are used in this manuscript:

FHD	Fréchet Histological Distance
FID	Fréchet Inception Distance
GAN	Generative Adversarial Network
GDPR	General Data Protection Regulation
HE	Hematoxylin and Eosin
IHC	Immunohistochemical
PPL	Perceptual Path Length
PSPStain	Pathological Semantics-Preserving Learning method for Virtual Staining

## Appendix A. Heatmap Tables for Ki67 Intervals

**Table A1:** FID heatmap results for dataset vs. generator Ki67 intervals.

			Generator					
			**<0.5,1>**	<0,0.5)				
					**<0.2,0.5)**	**<0,0.2)**		
**Dataset**							**<0.1,0.2)**	**<0,0.1)**
**<0.5,1>**			**15.16**	19.10	16.45	19.85	17.18	20.52
**<0,0.5)**			19.72	**5.67**	9.80	5.52	7.13	5.59
	**<0.2,0.5)**		20.50	8.17	**9.90**	8.42	9.45	8.57
	**<0,0.2)**		22.12	5.45	10.26	**5.59**	7.24	5.65
		**<0.1,0.2)**	19.53	6.52	8.76	6.59	**6.61**	7.01
		**<0,0.1)**	20.77	5.86	10.75	5.67	7.72	**5.64**

**Table A2:** FHD heatmap results for dataset vs. generator Ki67 intervals.

			Generator					
			**<0.5,1>**	<0,0.5)				
					**<0.2,0.5)**	**<0,0.2)**		
**Dataset**							**<0.1,0.2)**	**<0,0.1)**
**<0.5,1>**			**32.41**	43.08	33.39	45.68	37.62	47.95
**<0,0.5)**			45.40	**10.36**	19.85	10.41	12.95	10.83
	**<0.2,0.5)**		35.47	23.42	**20.71**	25.70	22.31	26.67
	**<0,0.2)**		44.97	10.49	21.05	**10.45**	13.70	10.77
		**<0.1,0.2)**	37.01	15.28	17.56	16.51	**13.07**	17.83
		**<0,0.1)**	47.78	11.32	23.86	11.14	15.31	**11.03**

**Table A3:** FHD heatmap results for dataset vs. dataset Ki67 intervals.

			Dataset					
			**<0.5,1>**	<0,0.5)				
					**<0.2,0.5)**	**<0,0.2)**		
**Dataset**							**<0.1,0.2)**	**<0,0.1)**
**<0.5,1>**			**0.00**	40.95	31.47	44.38	32.75	47.80
**<0,0.5)**			40.95	**0.00**	12.82	0.30	6.19	0.87
	**<0.2,0.5)**		31.47	12.82	**0.00**	16.81	9.43	19.56
	**<0,0.2)**		44.38	0.30	16.81	**0.00**	7.72	0.24
		**<0.1,0.2)**	32.75	6.19	9.43	7.72	**0.00**	10.58
		**<0,0.1)**	47.80	0.87	19.56	0.24	10.58	**0.00**

**Table A4:** FHD heatmap results for generator vs. generator Ki67 intervals.

			Generator					
			**<0.5,1>**	<0,0.5)				
					**<0.2,0.5)**	**<0,0.2)**		
**Generator**							**<0.1,0.2)**	**<0,0.1)**
**<0.5,1>**			**0.42**	33.87	13.11	38.12	29.10	41.70
**<0,0.5)**			33.87	**0.45**	9.99	0.70	3.27	1.05
	**<0.2,0.5)**		13.11	9.99	**0.44**	12.92	5.72	15.35
	**<0,0.2)**		38.12	0.70	12.92	**0.44**	4.41	0.61
		**<0.1,0.2)**	29.10	3.27	5.72	4.41	**0.42**	6.00
		**<0,0.1)**	41.70	1.05	15.35	0.61	6.00	**0.44**

**Table A5:** PPL heatmap results for Ki67 intervals.

<0,1>						
	<0.5,1>	<0,0.5)				
			<0.2,0.5)	<0,0.2)		
					<0.1,0.2)	<0,0.1)
1526.11	1561.95	1549.54	1496.20	1533.52	1482.50	1559.26
